# FasL Is Required for Osseous Healing in Extraction Sockets in Mice

**DOI:** 10.3389/fimmu.2021.678873

**Published:** 2021-05-31

**Authors:** Karol Alí Apaza Alccayhuaman, Patrick Heimel, Jung-Seok Lee, Stefan Tangl, Franz J. Strauss, Alexandra Stähli, Eva Matalová, Reinhard Gruber

**Affiliations:** ^1^ Department of Oral Biology, Medical University of Vienna, Vienna, Austria; ^2^ Karl Donath Laboratory for Hard Tissue and Biomaterial Research, School of Dentistry, Medical University of Vienna, Vienna, Austria; ^3^ Ludwig Boltzmann Institute for Experimental and Clinical Traumatology, Vienna, Austria; ^4^ Austrian Cluster for Tissue Regeneration, Medical University of Vienna, Vienna, Austria; ^5^ Department of Periodontology, Research Institute for Periodontal Regeneration, College of Dentistry, Yonsei University, Seoul, South Korea; ^6^ Clinic of Reconstructive Dentistry, University of Zurich, Zurich, Switzerland; ^7^ Department of Conservative Dentistry, School of Dentistry, University of Chile, Santiago, Chile; ^8^ Department of Periodontology, School of Dental Medicine, University of Bern, Bern, Switzerland; ^9^ Institute of Animal Physiology and Genetics, Czech Academy of Sciences, Brno, Czechia

**Keywords:** dentistry, bone regenaration, tooth extraction, fasl, knockout (KO), µCT, histology

## Abstract

Fas ligand (FasL) is a member of the tumor necrosis factor (TNF) superfamily involved in the activation of apoptosis. Assuming that apoptosis is initiated after tooth extraction it is reasonable to suggest that FasL may play a pivotal role in the healing of extraction sockets. Herein, we tested the hypothesis of whether the lack of FasL impairs the healing of extraction sockets. To this end, we extracted upper right incisors of FasL knockout (KO) mice and their wildtype (WT) littermates. After a healing period of two weeks, bone volume over total volume (BV/TV) *via* µCT and descriptive histological analyses were performed. µCT revealed that BV/TV in the coronal region of the socket amounted to 39.4% in WT and 21.8% in KO, with a significant difference between the groups (p=0.002). Likewise, in the middle region of the socket, BV/TV amounted to 50.3% in WT and 40.8% in KO (p<0.001). In the apical part, however, no difference was noticed. Consistently, WT mice displayed a significantly higher median trabecular thickness and a lower trabecular separation when compared to the KO group at the coronal and central region of the socket. There was the overall tendency that in both, female and male mice, FasL affects bone regeneration. Taken together, these findings suggest that FasL deficiency may reduce bone regeneration during the healing process of extraction sockets.

## Introduction

FasL (CD178; CD95L; APO1L and TNF ligand superfamily member 6) belongs to the tumor necrosis factor (TNF) family and interacts with Fas (CD95; APO-1; TNFRSF6) receptor (1). The FasL/Fas pathway is the common initiator of an extrinsic apoptotic machinery engaged in the immune system (2–4) having the potential to affect the development of teeth (5) and bones (6–8). Fas and Fas ligand are present in the jaw bone and tooth germs of human fetuses (9). Moreover, mice homozygous for the FasL point mutation display an osteopetrotic phenotype in their long bones (10). These mice even showed an enhanced bone formation upon stimulation with demineralized bone and BMP-2 when compared to wildtype mice (11, 12). FasL when expressed by osteoblast controls osteoclast apoptosis (13, 14), number and activity (15). Considering that bone regeneration involves the coordinated activity of osteoclasts and particularly of osteoblasts, there is reason to suggest that FasL is required for the healing of tooth extraction sockets.

Healing of extraction sockets has become an important issue in dentistry particularly due to the increasing demand of dental implants as a therapy to replace missing teeth (16). The alveolar bone usually undergoes atrophy upon the tooth extraction (17–19), and various treatment regimens were developed and introduced for extraction socket grafting. However, histologic bone quality differs with varying degrees of new bone formation (20). Therefore, there has been a great interest to understand bone regeneration and the healing process of extraction sockets. Bone regeneration is a sequential process of events that partially recapitulates bone development involving cell apoptosis and the coordinated action of osteoblast and osteoclasts (21–23). Accumulating evidence suggests that the FasL/Fas system is involved in bone regeneration. For example, mice lacking Fas show delayed cartilage resorption and less bone in the fracture calluses (24). With respect to wound healing, the lack of FasL/Fas signaling impairs apoptosis in granulation tissue and mononuclear cells (25, 26). Thus, it seems conceivable that FasL is involved in the healing of extraction sockets.

The aim of the present study was, therefore, to examine whether or not the lack of FasL impairs the healing of extraction sockets. To test this assumption, we took advantage of the established FasL knockout mice along with a recent established tooth extraction model in mice (27). Based on a segmentation of the alveolar socket, it is possible to measure new bone formation, that in combination with histology of undecalcified thin ground sections, provides insights into the overall healing situation of the extraction socket. Based on this approach, we identified FasL as a molecular target involved in the bone regeneration and healing process of extraction sockets in rodents.

## Material and Methods

### Study Design

The Medical University of Vienna ethical review board for animal research approved the study protocol (GZ BMWFW-66.009/0359-V/3b/2018). The study was performed at the Department of Biomedical Research of the Medical University of Vienna in accordance with the NC3Rs ARRIVE guidelines. Mice homozygous for the Fasl*gld* mutation (B6Smn.C3-Faslgld/J) were purchased from The Jackson Laboratory (Bar Harbor, ME) and housed in the Medical University of Vienna, Institute of Biomedical Research under specific-pathogen-free (SPF) conditions. FasL knockout mice and littermate controls (8-12 weeks, around 22 g) underwent tooth extraction of the upper right incisor. The animals were maintained according to the animal welfare guidelines with free access to water and a standard diet (28).

### Tooth Extraction Model

The tooth extraction model was performed as recently described (27). In brief, all animals received ketamine 100 mg/kg (AniMedica, Senden, Erlangen, Germany) and xylazine hydrochloride 5mg/kg (Bayer Austria, Vienna, Austria) by intramuscular injection. Then, the head of the mouse was stabilized by holding the contralateral tooth with a tweezer. Next, with the aid of a stereomicroscope (Leica M651, Leica Microsystems, Wetzlar, Germany) under 16X magnification, the upper right incisor was luxated using disposable needles (HSW FINE-JECT^®^, Tuttlingen, Germany) of different diameters (0.4 mm, 0.6 mm and 0.8 mm) as periotomes. After a proper luxation, the tooth was carefully extracted to avoid any root fracture using an Adson tweezer (Aesculap, Tuttlingen, Germany) and checked for integrity. For pain relief, buprenorphine 0.06 mg/kg s.c. (Temgesic^®^, Reckitt and Colman Pharm., Hull, UK) and piritramide in drinking water ad lib was administered. The first 72 hours after surgery soft diet was provided. Mice were euthanized on day fourteen by cervical dislocation and each alveolar socket was subjected to micro computed tomographic (µCT) and histological analysis.

### MicroCT Analysis

**Figure 1:**
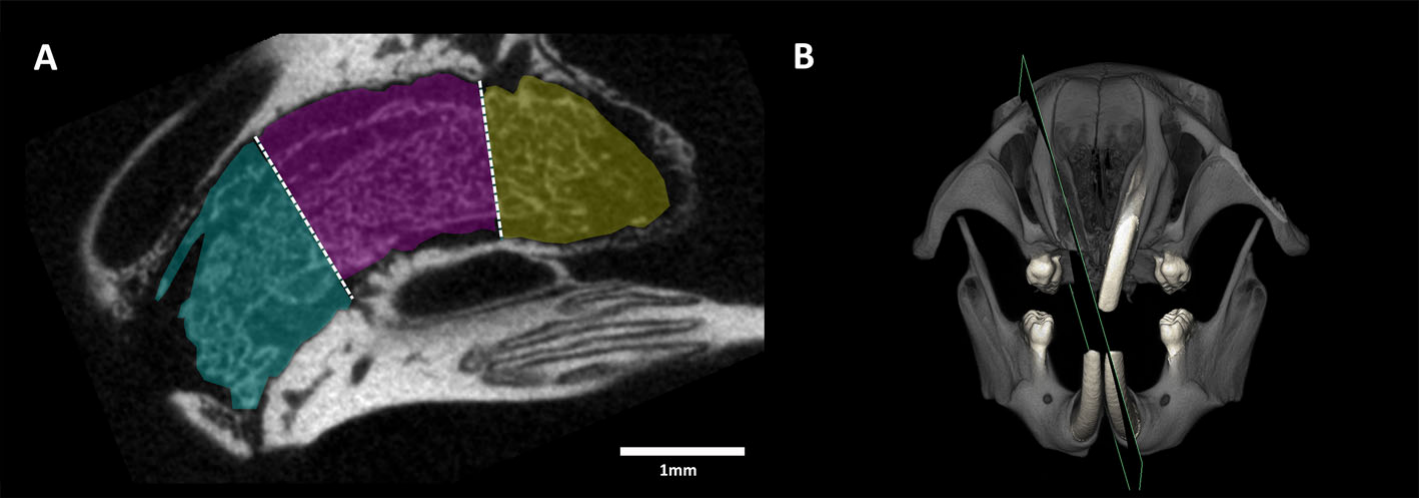
Region of interests (ROI) of the extraction socket for the µCT analysis. **(A)** The ROIs for the microCT analysis comprised the coronal (cyan), middle (purple) and apical (yellow) region through the entire volume of the tooth extraction site. **(B)** The plane oriented along the central alveolar socket was the reference for preparing the histological ground sections.

After euthanasia, the heads were fixed in phosphate-buffered formalin (Roti-Histofix 4%, Carl Roth, Karlsruhe, Germany). MicroCT scans were made using a Scanco µCT 50 (Scanco Medical AG, Bruttisellen, Switzerland) at 90 kV/200 µA with an isotropic resolution of 10 µm and an integration time of 500 ms. Using Amira 6.1.1 (Thermo Fisher Scientific, Waltham, USA), The image stacks were imported into Fiji for the posterior analysis (29, 30). The region of interest (ROI) was drawn using the polygon and freehand selection tools and saved using the ROI manager. To have a standardized position of the ROIs from all samples, four anatomical landmarks were set up, thereby dividing the alveolar socket in three regions (coronal, middle and apical) from the rearmost point to the most frontal point (**Figure 1A**). The percentage of Bone volume per Tissue volume (BV/TV), Trabecular thickness (Tb.Th) and Trabecular separation (Tb.Sp) were measured in the ROI with a threshold of 254 mgHA/cm³.

### Histological Analysis

Ten samples were dehydrated with ascending alcohol grades and embedded in light-curing resin (Technovit 7200 VLC + BPO; Kulzer & Co., Wehrheim, Germany). Blocks were further processed using Exakt cutting and grinding equipment (Exakt Apparatebau, Norderstedt, Germany). Thin-ground sections from all samples were prepared (31), in a plane parallel to the sagittal suture and through the middle of the alveolar socket and stained with Levai–Laczko dye (**Figure 1B**). The slices of around 100 µm were scanned using an Olympus BX61VS digital virtual microscopy system (DotSlide 2.4, Olympus, Tokyo, Japan) with a 20x objective resulting in a resolution of 0.32 µm per pixel and then evaluated.

### Statistical Analysis

Statistical analysis was based on the data obtained from the microCT analysis. Median values and confidence intervals (CI) of the primary endpoint, bone volume (BV/TV) and secondary endpoints (Tb.Th and Tb.Sp) in the alveolar socket, between the two groups were compared with Mann-Whitney U test. Further analyses were performed comparing the mice gender and intragroup between WT and FasL KO using the Mann-Whitney U test. All the analyses were performed using Prism v7 (GraphPad Software, La Jollia, CA). Significance was set at p<0.05.

## Results

### Micro CT Analysis of Bone Volume Per Tissue Volume (BV/TV)

**Figure 2:**
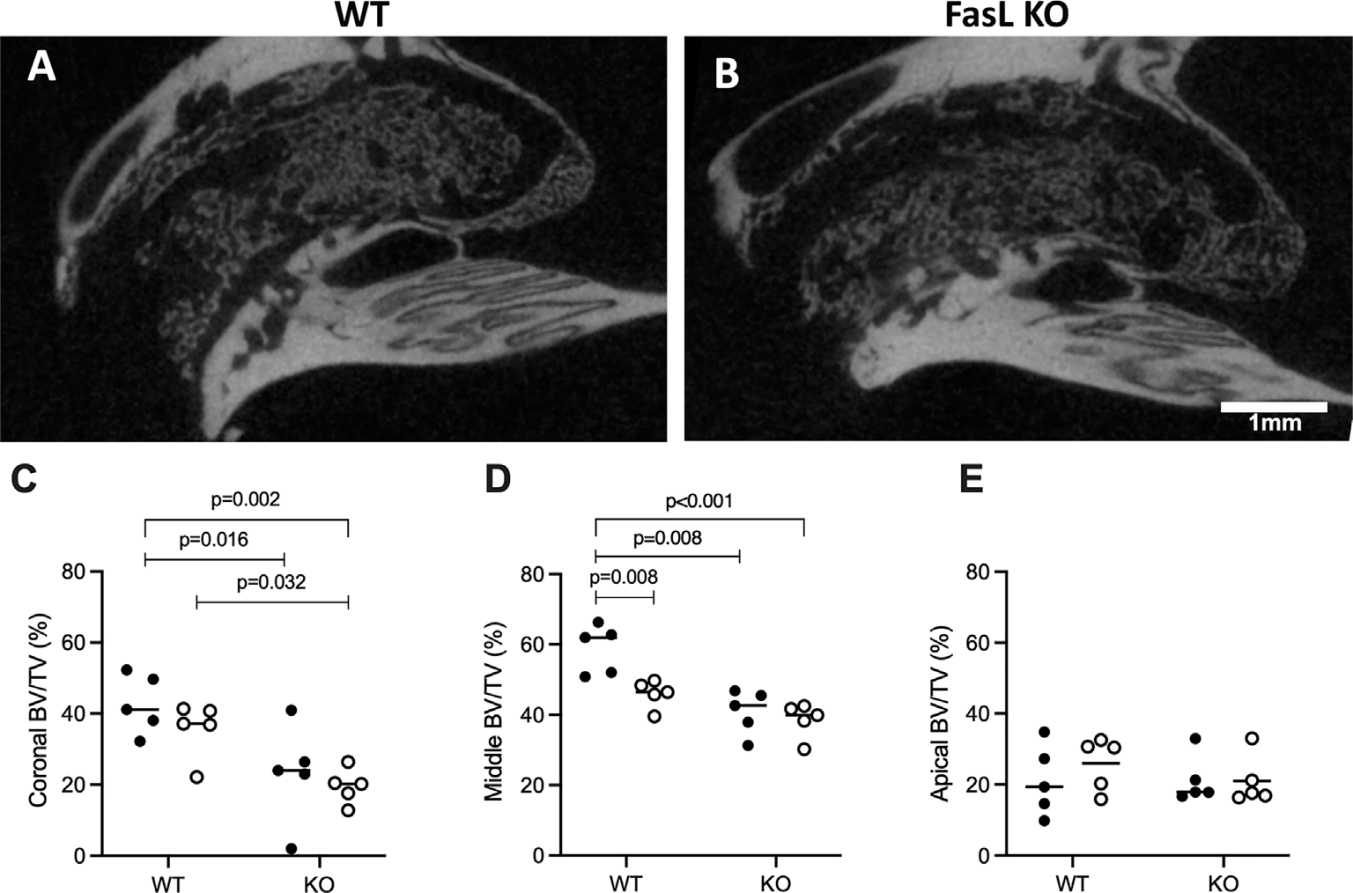
Lack of FasL attenuates regeneration of the extraction socket. Sagittal view of the alveolar socket depicts the WT **(A)** and FasL KO mice **(B)**. Quantitative analysis of the bone volume per tissue volume (BV/TV) displayed higher amounts of new bone volume in the WT mice in the coronal **(C)** and middle part **(D)** of the extraction socket compared to FasL KO mice. The apical region revealed no significant differences in BV/TV between WT and FasL KO mice **(E)**. Statistical analysis was based on Mann‐Whitney U test, P values are given where there was significant differences. The bars show the median and female (black dots) and male mice (white dots) are distributed in the dot plots for the WT and KO group.

For the analysis a total of ten WT and ten FasL KO mice were used comprising five males and females in each group (**Figure 2**). Statistical analysis revealed that the median BV/TV in the coronal region of the socket was significantly higher (p=0.002) in the WT than in the KO group, with 39.4% (22.1 min; 52.3 max) *versus* 21.8% (2.0 min; 41.0 max), respectively (**Figures 2A–C**). Likewise, in the middle region of the socket, the BV/TV in the WT was significantly higher (p<0.001) than in the KO group, with 50.3% (39.57 min; 66.31 max) and 40.8% (30.20 min; 46.81 max), respectively (**Figure 2D**). The median changes of BV/TV in the apical part of the socket failed to reach the level of significance (p=0.796; **Figure 2E**). Further analysis was performed comparing female and male mice. In the coronal and center region, the WT female (p=0.016; p=0.008) and WT male mice (p=0.032; p=0.056) displayed higher bone formation compared to the respective FasL KO mice. Intragroup comparisons revealed in the middle region a remarkable difference (p=0.008) between the male and female WT mice (**Figure 2D**), while in the other groups there were no gender differences. These findings suggest that FasL is involved in the formation of new bone volume in the extraction socket of female and male mice.

### Micro CT Analysis of Trabecular Thickness (TbTh) and Trabecular Separation (TbSp)

**Figure 3:**
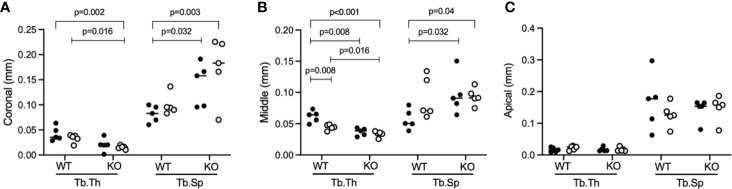
Trabecular thickness (TbTh) and trabecular separation (TbSp) in the extraction sockets. FasL KO mice shows lower Tb.Th and higher Tb.Sp compared to WT mice in the coronal **(A)** and middle part **(B)** while no differences were observed in the apical part **(C)**. Statistical analysis was based on Mann‐Whitney U test, P values are given where was found significant differences. The bars show the median with female (black dots) and male mice (white dots) are distributed in the dot plots.

In support of the bone volume fraction, the median trabecular thickness (TbTh) in the coronal region of the socket was significantly (p=0.002) higher in WT than in KO, with 0.04 mm (0.02 min; 0.06 max) *versus* 0.02 mm (0.002 min; 0.04 max), respectively (**Figure 3A**). This significant difference (p<0.001) was also observed in the middle part of the socket, with 0.05 mm (0.04 min; 0.07 max) in WT and 0.04 mm (0.03 min; 0.04 max) in KO, respectively (**Figure 3B**) but not in the apical part of the socket (**Figure 3C**). Consistently, coronal trabecular separation (TbSp) was significant higher (p=0.003) in WT than in KO mice, with 0.09 mm (0.06 min; 0.14 max) and 0.17 mm (0.07 min; 0.23 max), respectively (**Figure 3A**). Similarly, this difference was also significant (p=0.004) in the middle part, 0.07 mm (0.04 min; 0.13 max) *versus* 0.09 mm (0.06 min; 0.15 max), respectively (**Figure 3B**). In the apical part, nonetheless, there were no significant differences (**Figure 3C**). Considering mouse gender, there was the overall tendency that female and male mice, were similarly affected by the lack of FasL in the coronal and middle part of the extraction sockets (**Figures 3A, B**). Intragroup comparisons revealed that WT and KO female mice when compared to their male littermates, have a denser trabecular network reaching the level of significance in the middle region (**Figure 3B**). Taken together, these observations indicate that FasL is partially required for the proper formation of trabecular structures in both genders.

### Histological Analyses

**Figure 4:**
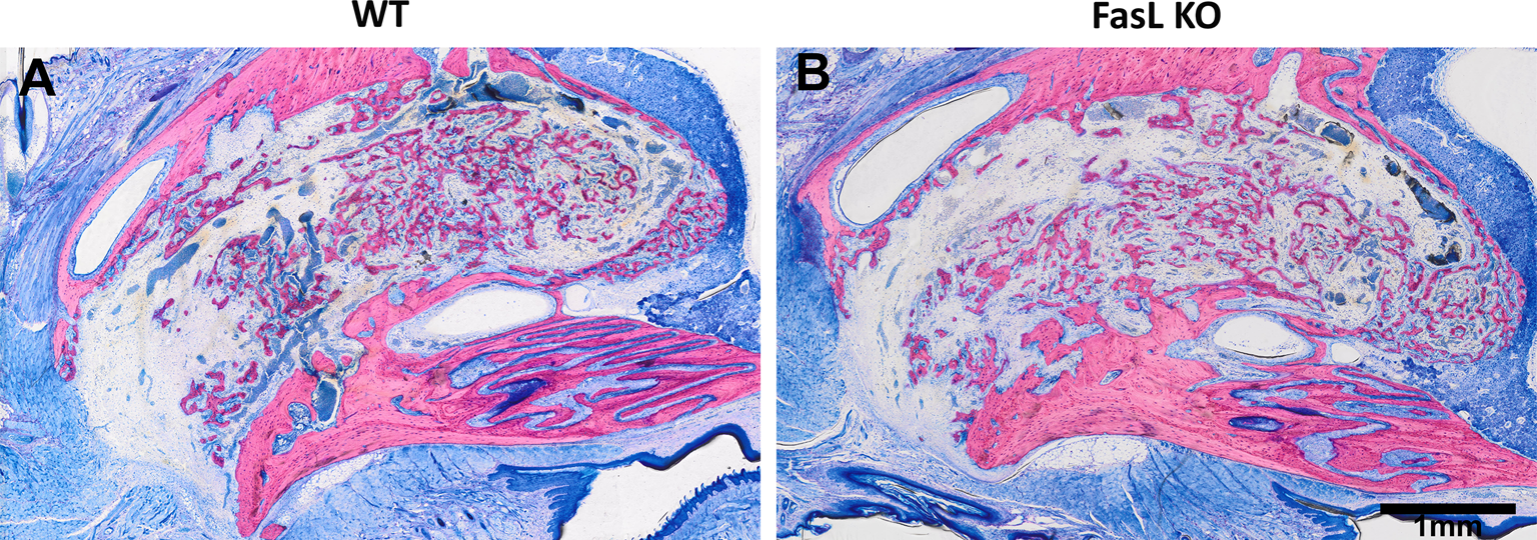
The features of the newly formed bone were similar for the WT **(A)** and the FasL KO mice **(B)**. Overview photomicrographs (2x) depicting the woven network.

**Figure 5:**
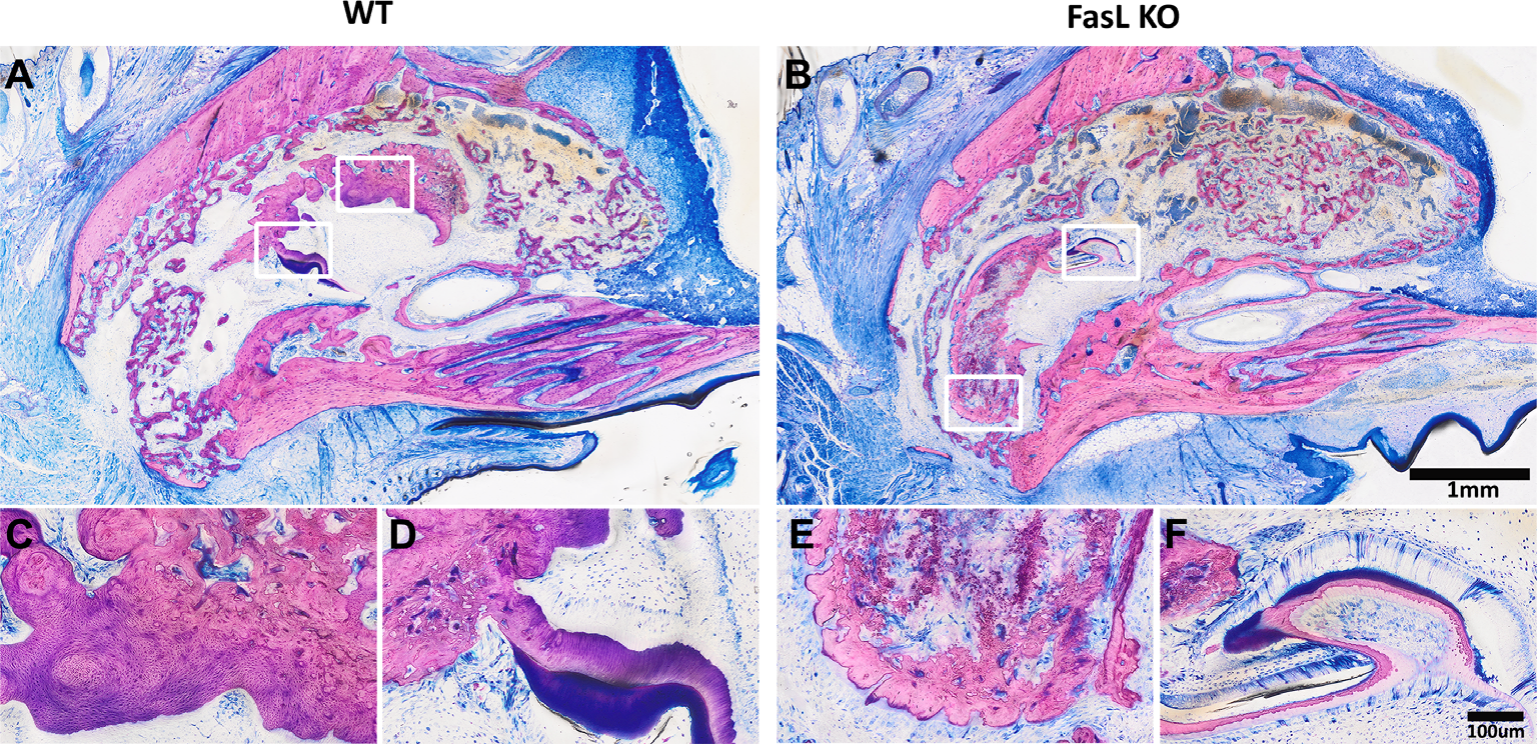
The WT **(A)** and The FasL KO **(B)** mice revealed a structure like a growing tooth located at different areas of the alveolar socket in the overview image (2x). A higher magnification (20x) evidenced the “osteodentin mass” with dentin tubules **(C, E)** and enamel structure **(D, F)**.

Newly generated woven bone was observed in the WT and FasL KO mice (**Figure 4**). This woven bone formed a trabecular network with random orientation surrounded either by thin layers of parallel fibered bone or thin layers of unmineralized matrix osteoid. Newly formed bone was located next to the coronally bone walls and in the middle, filling almost completely the alveolar defect, while the FasL KO mice exhibited a trabecular bone enclosing bigger spaces formed between. We also observed signs of growing teeth denoted by the formation of new dentine and enamel in both, the FasL KO and WT mice (**Figure 5**). These structures, nevertheless, were excluded from the µCT analysis by segmentation.

## Discussion

The present pre-clinical study revealed that the lack of FasL attenuates the healing process of extraction socket in female and male mice. This research was inspired by the fact that FasL is a central regulator of apoptosis (2–4) having a major impact on bone cells life cycle thereby affecting osteoblasts and osteoclasts during bone remodeling. FasL is a key target for estrogen to control osteoblast-mediated osteoclast apoptosis (13–15). FasL was reported to delay cartilage resorption and bone formation in the fracture calluses (24) and wound healing. Moreover, FasL/Fas signaling controls apoptosis in granulation tissue and mononuclear cells (25, 26). To the best of the authors knowledge, the impact of FasL on bone regeneration, and in particular the intramembranous ossification upon tooth extraction inside the remaining alveolus, had not been investigated. Assuming that dying cells upon tooth extraction are presumably responsible for triggering the signals for repair and regeneration, we raised the hypothesis that the lack of FasL impairs the overall capacity of bone regeneration. Hereby we report that female and male mice deficient in FasL, show less bone formation in extraction sockets as compared to WT mice. This finding supports the notion that FasL is required for the bone healing process of extraction sockets.

If we compare the present findings to those of others, our data are in line with the critical involvement of FasL in bone formation. For example, embryonic and early 6-week postnatal FasL knockout mice showed less mandibular and alveolar bone compared to WT littermates (32, 33). Consistently, in 22-week old mice, whole genome FasL KO mice have less trabecular and cortical bone in the axial and appendicular skeleton compared to their WT littermates (34). Even osteoblast progenitor/osteoblast-specific FasL-deficient mice showed markedly reduced bone density and structural parameters in the femurs (15). Our findings that FasL knockout mice showed less bone formation in the alveolar socket upon tooth extraction compared to the WT littermates are consistent with the relevance of FasL in bone formation. Furthermore, these findings are largely in agreement with the observation that already after two weeks healing, the extraction socket of incisors is almost completely filled with an immature woven bone-like tissue (27, 35).

The present study has a number of limitations. In the knockout mice, FasL is missing in all cells, meaning that we cannot draw conclusions whether or not the observed effects are caused by osteoblast or osteoclasts. It is plausible that the lack of FasL affects angiogenesis, implying that the impaired bone regeneration observed in the KO mice, is rather a consequence of a reduced supply of osteogenic progenitor cells (36). Future studies should therefore take advantage of conditional knockout models where FasL is deleted in specific cells types, for example with osteoblast progenitor/osteoblast-specific FasL-deficient mice (15). Thus, the origin of FasL capable of driving the FasL-dependent apoptosis- or other apoptosis-independent responses during bone regeneration remains to be elucidated. Another study limitation was that we could not avoid sites with new tooth formation. The present findings might also serve for researchers asking if overexpression of FasL can support bone regeneration, for example by implementing the respective agonists (37) or using a transgenic FasL mouse model (38). It would also be interesting to know why female compared to male WT mice, show more a pronounced healing of extraction sockets. This was unexpected since male muscle-derived stem cells regenerated more bone than female cells in a calvaria defect (39). Nevertheless, the estrogen receptor beta is required for proper healing in female mice that however, does not rule out that it is also required in male mice (40). Moreover, ovariectomy impairs bone formation in drill-hole defects, but this setting does not allow a gender comparison (41). Hence, future research could focus more on the impact of gender on the healing of extraction sockets.

Future research should further use the FasL model to investigate fracture healing, as the impact of FasL on endochondral bone formation is not necessarily reflected by the tooth extraction model of intramembranous ossification that was used (21, 23). In fact, it is unclear whether a tooth extraction model represents intramembranous ossification in other anatomical regions that are perhaps more representative for the appendicular skeleton, beyond the field of dentistry. Our model, however, seems to be suitable to better understand the biology of the healing of extraction sockets and presumably also the osseointegration of dental implants that follow the same conserved sequence of events (42). It can be speculated that targeting the FasL system could be a therapeutic option to boost osseointegration, nevertheless, this would require data showing that pushing physiological FasL signaling enhances the bone regeneration (37). Certainly, the present research is a further step towards FasL-signaling in bone regeneration.

In conclusion, these data suggest that FasL is required for bone regeneration during the healing process of tooth extraction sockets.

## Data Availability Statement

The original contributions presented in the study are included in the article/supplementary material. Further inquiries can be directed to the corresponding author.

## Ethics Statement

The animal study was reviewed and approved by Austrian Federal Ministry of Education, Science and Research.

## Author Contributions

RG and EM contributed to the ​conception and design of the study. KAAA and PH work on the measurements of the data and organized the database. RG and KAAA wrote the first draft of the manuscript. FS, ST, and JL wrote sections of the manuscript. All authors contributed to the article and approved the submitted version.

## Funding

This research project is funded by a grant from the Austrian Science Fund (FWF) (4072‐B28) joint with the Czech Science Foundation (GACR) (19-29667L). KAAA is supported by an Osteology Research Scholarship.

## Conflict of Interest

The authors declare that the research was conducted in the absence of any commercial or financial relationships that could be construed as a potential conflict of interest.
